# Altered resting-state functional connectivity in women survivors of intimate partner violence: an ICA study

**DOI:** 10.1186/s40359-026-04063-x

**Published:** 2026-03-20

**Authors:** María Pérez-González, María Dolores Sánchez-Rodríguez, Julia C. Daugherty, Natalia Hidalgo-Ruzzante, Miguel Pérez-García, Sofia Amaoui, Juan Verdejo-Román

**Affiliations:** 1 Department of Personality, Assessment and Psychological Treatment, University of Granada, Granada, 18011 Spain; 2 Mind, Brain and Behavior Research Center at University of Granada, Granada, 18011 Spain; 3 Laboratory of Social and Cognitive Psychology, University of Clermont Auvergne, Clermont-Ferrand, Auvergne-Rhône-Alpes 63000 France; 4 Department of Developmental and Educational Psychology, University of Granada, Granada, 18011 Spain; 5 Department of Psychology, University of Innsbruck, Innsbruck, 6020 Austria

**Keywords:** Domestic violence, Gender-based violence, fMRI, Brain networks, Independent component analysis, Default mode network, Dorsal attention network, Anxiety, Depression, PTSD

## Abstract

**Background:**

This study examines differences in large-scale resting-state networks (RSNs) connectivity between women who survived intimate partner violence (IPVAW) and non-victims. It also explores the association between specific resting-state functional connectivity (rs-FC) patterns in IPVAW survivors and both the severity of experienced violence and relevant clinical symptoms, including depression, anxiety, and posttraumatic stress disorder (PTSD).

**Methods:**

Participants underwent resting-state fMRI and completed self-report assessments on the severity of the IPVAW experience, adverse childhood experiences, PTSD, anxiety, depression, and alcohol use. Independent Component Analysis (ICA) was used to characterize RSNs. Between-group differences were examined using a T-test while controlling for age and level of education. Exploratory correlations were conducted to examine associations between the rs-FC patterns in IPVAW survivors and the severity of violence and clinical measures.

**Results:**

IPVAW survivors showed less rs-FC within the default mode network (DMN) and dorsal attention network (DAN). They also showed greater rs-FC of the cerebellum with the posterior DMN, salience network (SN), and posterior sensorimotor network (pSMN) compared to non-victims. Specific rs-FC patterns in IPVAW survivors were significantly associated with clinical symptoms.

**Conclusions:**

The findings indicate that IPVAW survivors show specific intrinsic functional connectivity that is associated with psychopathological symptoms. The present study contributes to a deeper understanding of these neural correlates and may support intervention programs aimed at addressing long-term sequelae of IPVAW.

**Supplementary Information:**

The online version contains supplementary material available at 10.1186/s40359-026-04063-x.

## Background

One out of three women worldwide has suffered physical and/or sexual violence by their partner or ex-partner at some point in her lifetime [1]. Furthermore, it has been estimated that 38.6% of female homicides in the world are caused by intimate partner violence [1].

Studies on the consequences of intimate partner violence against women (IPVAW) have indicated that women survivors often experience a range of physical issues such as chronic health conditions, neuroendocrine and immune disorders [2–5] as well as psychological challenges including anxiety, depression, and post-traumatic stress disorder (PTSD) [6–12]. Recent literature has demonstrated that women survivors of IPVAW also experience cerebral consequences such as traumatic brain injury (TBI), structural brain alterations [13–17] and neuropsychological alterations in verbal memory, working memory, cognitive flexibility, processing speed, attention, and inhibition domains [16, 18–25].

Building on this evidence, research has turned towards examining the brain’s intrinsic functional organization - how different brain regions interact in the absence of external stimuli - to better understand the neural underpinnings associated with experiencing IPVAW [26, 27]. A common method for investigating this intrinsic connectivity is resting-state functional magnetic resonance imaging (rs-fMRI), which measures spontaneous fluctuations in the blood oxygen level-dependent (BOLD) signal. Through resting-state functional connectivity (rs-FC) analyses, researchers examine how temporally correlated BOLD signals reveal synchronized activity across brain regions, shedding light on the brain’s functional networks at rest [28]. Quantifying rs-FC can be achieved through rigorous analytical methods, including independent component analysis (ICA) [29]. This analysis adopts a whole-brain exploratory approach by decomposing the data into independent components that correspond to neural networks (RSNs) enabling the study of both intra- and inter- network connectivity across the brain [30–32].

Despite growing interest in rs-FC in populations that have suffered different types of violence [33–37], only two studies have applied this approach to IPVAW survivors [15, 16], leaving much to be uncovered about the intrinsic neural alterations specifically associated with this violence. In particular, Valera & Kucyi [16] found that connectivity between the anterior insula—a core region of the salience network (SN)—and the precuneus, a key node of the default mode network (DMN) was negatively associated with the severity of the TBI resulting from IPVAW. Similarly, Likitlersuang [15] observed reduced connectivity between the isthmus cingulate and frontoparietal regions in IPVAW survivors suffering from TBI and PTSD. Notably, these two studies reflect altered inter- network connectivity rather than connectivity between regions within the same network. In related research, Roos and colleagues [38] performed a whole-brain graph approach that showed altered regional and global structural connectivity in IPVAW survivors —specifically involving the anterior cingulate cortex (a key SN region), the middle temporal gyrus (primarily associated with the DMN), and several subcortical structures—compared to the non-victim control group.

Although these studies have provided valuable insights into the intrinsic neural architecture of women who have survived IPVAW, no research to date has comprehensively examined network-level organization using a data-driven, large-scale functional network analysis in this population. Additionally, unlike prior studies that have primarily focused on specific subgroups of survivors—such as those with versus without TBI [15] or IPVAW survivors without PTSD recruited from alcohol abuse programs [38], there is a lack of research examining intrinsic brain connectivity differences in a more heterogeneous and representative IPVAW population, with comparative designs that include non-victimized controls. Moreover, given the existing literature that has demonstrated that clinical symptoms highly prevalent in our population of interest - such as PTSD [39], anxiety disorders [40], and depression [27] - are related to rs-FC alterations, it remains essential to explore the association between rs-FC patterns and clinical symptoms in IPVAW survivors.

To advance the understanding of intrinsic connectivity alterations in survivors of IPVAW, the primary aim of this study was to compare, for the first time, large-scale RSNs between women survivors of IPVAW and non-victims. Based on previous research in rs-FC, we focused on the default mode network (DMN) due to its involvement in self-referential and social cognition [41], the salience network (SN) for its role in stimulus detection [42], the frontoparietal network (FPN) given its contribution to cognitive control and working memory [43], and the dorsal attention network (DAN) because of its importance for goal-directed attention [43]. We further extended the scope to the sensorimotor network (SMN) associated with bodily awareness and pain processing [44], given the relevance of these domains to IPVAW-related symptomatology [45].

As an exploratory aim, this study investigates the associations between specific rs-FC patterns in IPVAW survivors and both the severity of experienced violence and relevant clinical variables, including depression, anxiety, and PTSD symptoms. Examining whether clinical symptoms and violence severity relate to atypical network connectivity could help clarify how IPVAW sequelae map onto changes in large-scale brain networks. This objective could also guide future hypothesis-driven research on symptom-linked neural alterations in IPVAW survivors.

Because this is the first study to use an ICA-based approach to examine RSNs in women survivors of IPVAW, we refrain from establishing specific connectivity-related hypotheses. We only expect that IPVAW survivors will show differences in rs-FC across the networks compared to non-victims. Likewise, we expect that the specific connectivities observed in IPVAW survivors will be associated with the severity of the experienced violence and the clinical symptoms (i.e., PTSD, depression, and anxiety).

## Methods

### Participants

A total of 78 women, aged 18 to 68, participated in the study. Half of the participants (*n* = 39) had experienced IPVAW and were recruited from women’s information centers affiliated with the Andalusian Women’s Institute. The remaining 39 participants, who had not experienced IPVAW, were recruited via word-of-mouth referrals as a non-victim group. Participants in this study correspond to those described in Pérez-González et al. [46], and inclusion and exclusion criteria can be found in supplementary material. Main sociodemographic and clinical characteristics of participants can be found in Table 1.

**Table 1:** Sociodemographic and clinical characteristics of the survivors and Non-victims group

Variable	Survivors (*n* = 39)	Non-victims (*n* = 39)	*p*-value / χ²
Age [mean(*SD*)]	42.5 (11.6)	41.1 (14.7)	0.658
Education level [n *(%)*]			
Primary school	14 (35.9%)	3 (7.7%)	0.007**
Vocational and/or High-school	11 (28.2%)	12 (30.8%)	
University	14 (35.9%)	24 (61.5%)	
Nationality [n *(%)*]			
Spanish	33 (84.6%)	36 (92.3%)	0.288
Non-spanish	6 (15.4%)	3 (7.7%)	
CAS-SF-MAX [mean(SD)]	34.7 (15.0)	0 (0)	< 0.001***
Physical violence [n *(%)*]	33 (84.6%)	0 (0%)	
IPV-TBI [n *(%)*]			
No-TBI	16 (51.6%)	39 (100%)	
At least one IPV-TBI	15 (48.4%)	0 (0%)	
Mild TBI	15 (48.4%)	0 (0%)	
Severe TBI	5 (16.1%)	0 (0%)	
ACE [mean(*SD*)]	1.79 (1.85)	0.744 (1.23)	0.005**
PCL-5 [mean(*SD*)]	42.45 (18.8)	7.92 (12.4)	< 0.001***
GAD-7 [mean(*SD*)]	13.1 (5.85)	4.19 (3.66)	< 0.001***
PHQ-9 [mean(*SD*)]	14.9 (9.23)	4.32 (3.52)	< 0.001***
AUDIT [mean(*SD*)]	3.68 (3.22)	4.85 (3.88)	0.158

*:p-value < 0.05, ** *p* < .01, ***:*p* < .001. *ACE* Adverse Childhood Experiences, *CAS-SF-MAX* Maximum score obtained in Composite Abuse Score, *GAD-7* Generalized Anxiety Disorder, *PCL-5* PTSD checklist for DSM-5, *PHQ-9* Patient Health Questionnaire Depression Subscale. Continuous variables show mean and standard deviation values. For the clinical variables (i.e., ACE, PCL-5, GAD-7, PHQ-9, and AUDIT), data were missing for 2 to 4 participants per measure except for TBI where data were missing for 8 participants. The IPVAW survivors who suffered severe TBI are also included in the mild TBI category (see supplementary material for more details)

### Material

#### Violence and mental health assessment

A thorough assessment was carried out using the following questionnaires: (1) The Composite Abuse Scale Revised-Short Form (CASR-SF; [47]) to evaluate the highest severity of IPVAW suffered across the lifespan (to this end, the authors created a new variable called CASR-SF-MAX); (2) the Adverse Childhood Experiences Questionnaire (ACE; [48]) to examine adverse childhood events; (3) the Brain Injury Severity Assessment (BISA; [49]) for the evaluation of potential TBI; (4) the PTSD Checklist (PCL-5; [50]) for assessing PTSD; (5) the Generalized Anxiety Disorder Questionnaire (GAD-7; [51]) for generalized anxiety; (6) the Patient Health Questionnaire Depression Subscale (PHQ-9; [52]) for depression; and (7) the Alcohol Use Disorders Identification Test (AUDIT-C; [53]) for measuring alcohol consumption. Details about the structure of each questionnaire, including the number of items, response categories, and scoring are provided in the supplementary material.

### Procedure

This study is part of a larger initiative called “The Believe Project”, accessible at www.projectbelieve.info. The broader project focuses on evaluating the neuropsychological, cerebral, and mental health characteristics of women who have survived IPVAW through a series of evaluation sessions. Initially, participants took part in clinical and social interviews and completed questionnaires to assess their mental health status, experiences of TBI, and the severity of the IPVAW they suffered in a calm and private office environment (in the research laboratory and in women’s centers). The neuroimaging assessment took place during the second session at the Mind, Brain, and Behavior Research Center (CIMCYC) at the University of Granada. Both sessions were conducted by researchers who are experts in the field of clinical psychology.

Prior to the clinical and social interview and the MRI session, all participants received a comprehensive briefing on the study’s procedures and potential risks, with both verbal and written explanations provided. They were informed of the anonymity and confidentiality standards, and each participant signed an informed consent form to confirm their agreement to participate. Participants received a payment of 30 euros as compensation for their involvement. The study was approved by the Ethics Committee at the University of Granada (Spain), reference number 789/CEIH/2019.

### Design and data analysis

#### Image acquisition and preprocessing

The resting-state scan lasted 6 min and participants were instructed to keep their eyes closed, to relax, to avoid thinking about anything specific, and to not move or fall asleep during the whole session. The data were collected on a 3.0 T MRI scanner (Siemens TRIO) using a 32-channel head coil. During the acquisition, functional brain images were acquired using a T2*-weighted EPI (echo planar imaging) sequence with the following parameters: repetition time (TR) = 2.0 s; echo time (TE) = 25 msec; field of acquisition (FOV) = 238 × 238; slice thickness = 3.5 cm; acquisition matrix = 68 × 68; number of slices = 35; voxel size = 3.5 × 3.5 × 3.5 × 3.5 mm, 180 whole-brain volumes. A sagittal three-dimensional T1 weighted turbo-gradient-echo sequence was also obtained with the given parameters: TR = 2300 ms; TE = 3.1 ms; FOV = 256 × 256; slice thickness = 0.8 mm, acquisition matrix = 320 × 320, number of slices = 208; voxel size = 0.8 × 0.8 × 0.8. The anatomical image allows for the assessment of gross anatomical abnormalities and was used during preprocessing to improve normalization of the functional data.

Brain images were analysed using CONN, Functional Connectivity Toolbox v20.b (Massachusetts Institute of Technology, Cambridge, Massachusetts, USA), under Matlab R2020a (MathWorks, Natick, MA). Preprocessing steps included: (a) realignment of functional images; (b) slice-timing correction, (c) outlier detection using ART toolbox, (d) segmentation of structural and functional data, (e) coregistration of images using each participant’s anatomical scan, (f) normalization in the Montreal Neurological Institute (MNI) space and resampled to 2 mm isotropic voxel, (g) spatial smoothing with a Gaussian kernel of 6-mm full-width-at-half-maximum (FWHM). In addition, functional data were denoised, which included the regression of confounding effects using the CompCor strategy. This step incorporated 5 principal components from the WM and CSF, 12 motion regressors, regressors of noise components (one for each identified outlier scan during the outlier identification step), and 2 regressors of effect of rest, followed by a bandpass frequency filter between 0.008 and 0.09 Hz and linear detrending term. One participant was excluded from the analysis due to excessive head movement, resulting in less than 4 min of usable resting-state data [54].

#### Independent Component Analysis (ICA)

ICA was performed to analyze the different brain networks. This approach helps to identify temporally coherent components (ICs) that correlate with each other by estimating spatially independent regions [55]. Spatial ICA was run in the CONN toolbox v20.b using FastICA for estimation of components and GICA 1 back-projection for individual subject level spatial map. Dimensionality reduction was set to 64 and the number of ICs was set to 30 [56]. ICs were identified by using correlational spatial match-to-template approach in CONN supported by visual inspection. The RSNs of interest included: the Default mode network (DMN), Salience network (SN), Frontoparietal network (FPN), Dorsal attention network (DAN) and Sensorimotor network (SMN).

#### Between-group differences in ICA-derived resting-state functional connectivity

In order to investigate differences in rs-FC between the groups, T-tests were performed including age and education as control variables. Minimum threshold extents for all the fMRI analyses were estimated for multiple comparisons by Monte Carlo simulations using AlphaSim within the RESTplus V 1.2. Input parameters included the whole brain mask (242545 voxels), a cluster connection radius of 5 mm, and the actual smoothness of data after model estimation. Results were significant at an alpha value of 0.05 and an individual voxel threshold probability of *p* < .001. As a result, the minimum cluster size needed to consider the results significant was different for each component: 86 voxels for the anterior DMN, 81 voxels for the posterior DMN, 84 voxels for the DAN, 80 voxels for the SN, 88 voxels for the primary SMN, 85 voxels for the secondary SMN and 83 voxels for the FPN.

Afterwards, significant between-group connectivity differences were classified as inside or outside each network. Whole-sample intrinsic connectivity masks were generated for each network by summing the activation and deactivation maps from both groups, thereby obtaining a complete intrinsic connectivity map across the sample. Masks were generated using Statistical Parametric Mapping software [57]; Wellcome, Department of Cognitive Neurology, Institute of Neurology, Queen Square, London, Great Britain). Significant between-group connectivity differences were then overlapped with these whole-sample maps separately for each IC and labelled as ‘inside connectivities’ if they fell within the mask or ‘outside connectivities’ if they fell outside it.

#### Statistical analysis

JASP statistical software [58] was used to perform T-tests or Chi-square tests (depending on the variable) to examine between-group differences in sociodemographic and clinical variables.

In order to study whether the specific rs-FC patterns of IPVAW survivors were related to the clinical tests, partial Pearson correlations were conducted between the connectivity values extracted for each participant from regions showing significant between-group rs-FC differences and clinical variables (GAD-7, PHQ-9, PCL-5 total score, PCL-5 A, PCL-5B, PCL-5 C, PCL-5D, and CAS-SF-MAX), also controlling for age and educational level. In line with the exploratory aim of this study, correlation analyses were conducted with and without Bonferroni correction, to allow for the identification of preliminary patterns that may inform future hypotheses.

#### Follow-up analyses: controlling for adverse childhood experiences

Considering the scores on the ACE questionnaire were significantly higher for the IPVAW group (Table 1), and the well-established association between adverse childhood experiences and brain alterations in adulthood [59], the fMRI analyses were repeated to explore whether the observed significant results might be influenced by ACEs. In this follow-up analysis, ACE scores were included as an additional covariate alongside age and education. Additionally, the same approach was applied to the clinical-connectivity correlations by including ACE scores as a third covariate.

## Results

### Network characterization

**Fig. 1:**
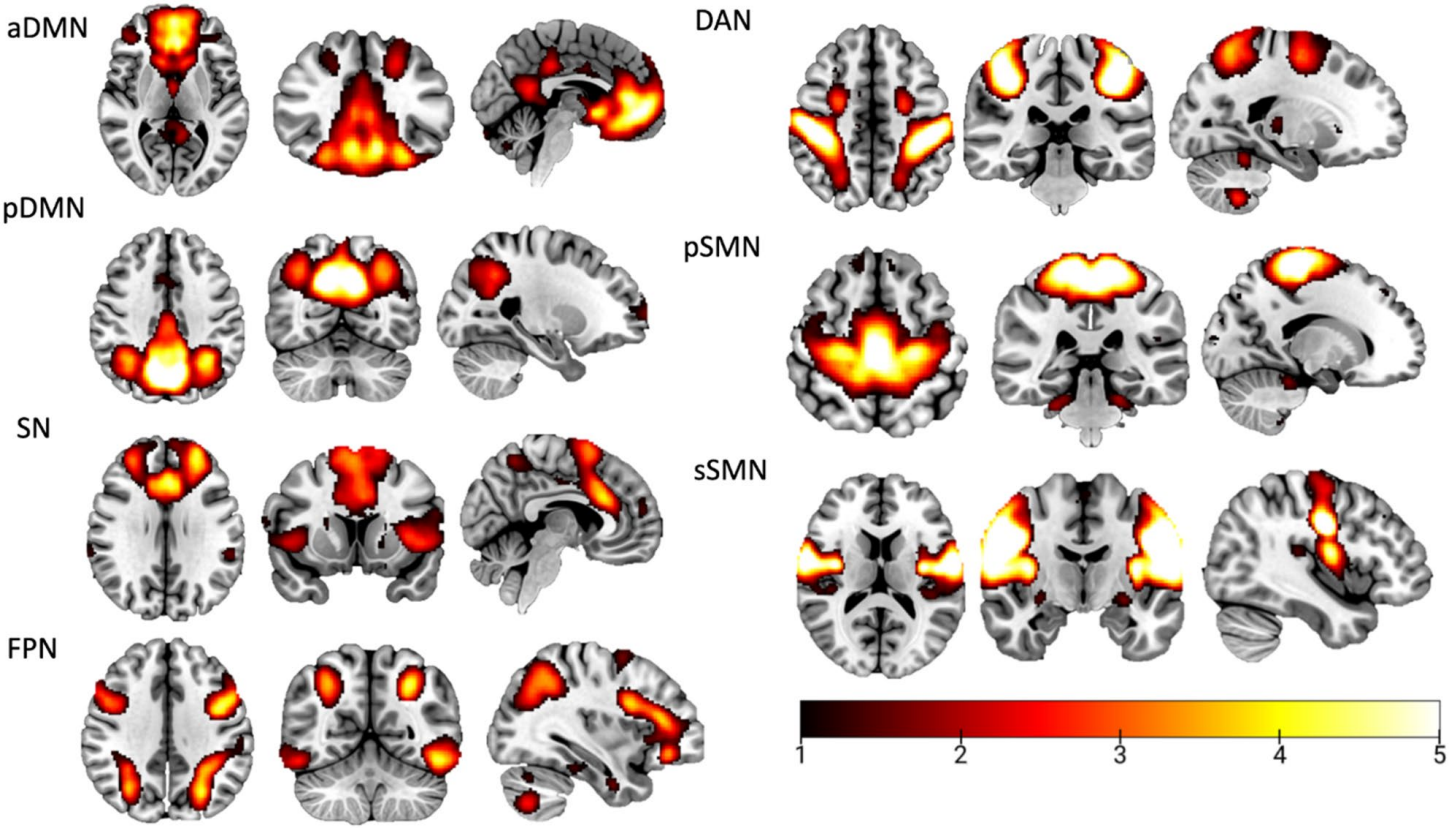
Spatial maps of the resting-state networks. Note. aDMN: Anterior default mode network, pDMN: Posterior default mode network, SN: Salience network, FPN: Frontoparietal network, DAN: Dorsal attention network, pSMN: Primary sensorimotor network, sSMN: Secondary sensorimotor network

Aligned with our objectives, a total of seven ICs were identified. Most networks were represented by a single IC, except for the DMN and the SMN. Specifically, the DMN was found divided into two components: one comprising the anterior subnetwork (aDMN) and another the posterior subnetwork (pDMN). Similarly, the SMN was found split into two components, representing the primary (pSMN) and secondary (sSMN) subnetworks. See Table 2; Fig. 1 for a more detailed characterization of IC and its respective network.

**Table 2:** Descriptive information of ICA-derived components

ICs	Functional label	Anatomical labels
1	Anterior DMN	Medial prefrontal cortex, ventral part of the anterior cingulate cortex, bilateral caudate, inferior and superior frontal gyrus, and hippocampus.
2	Posterior DMN	Precuneus, posterior cingulate cortex and bilateral angular gyrus.
3	Salience	Dorsal anterior cingulate, fronto-insular cortex.
4	Frontoparietal	Intraparietal sulcus, left and right dorsolateral prefrontal cortex and parietal lobes.
5	Dorsal Attention	Bilateral intraparietal sulcus and the frontal eye fields extending to the precentral gyrus.
6	Primary Sensorimotor	Pre-central and post-central sulcus, and the supplementary motor area.
7	Secondary Sensorimotor	Pre-central and post-central sulcus.

*DMN* Default mode network

### Between-group differences in ICA-derived rs-FC and association with clinical and violence-related variables

**Fig. 2:**
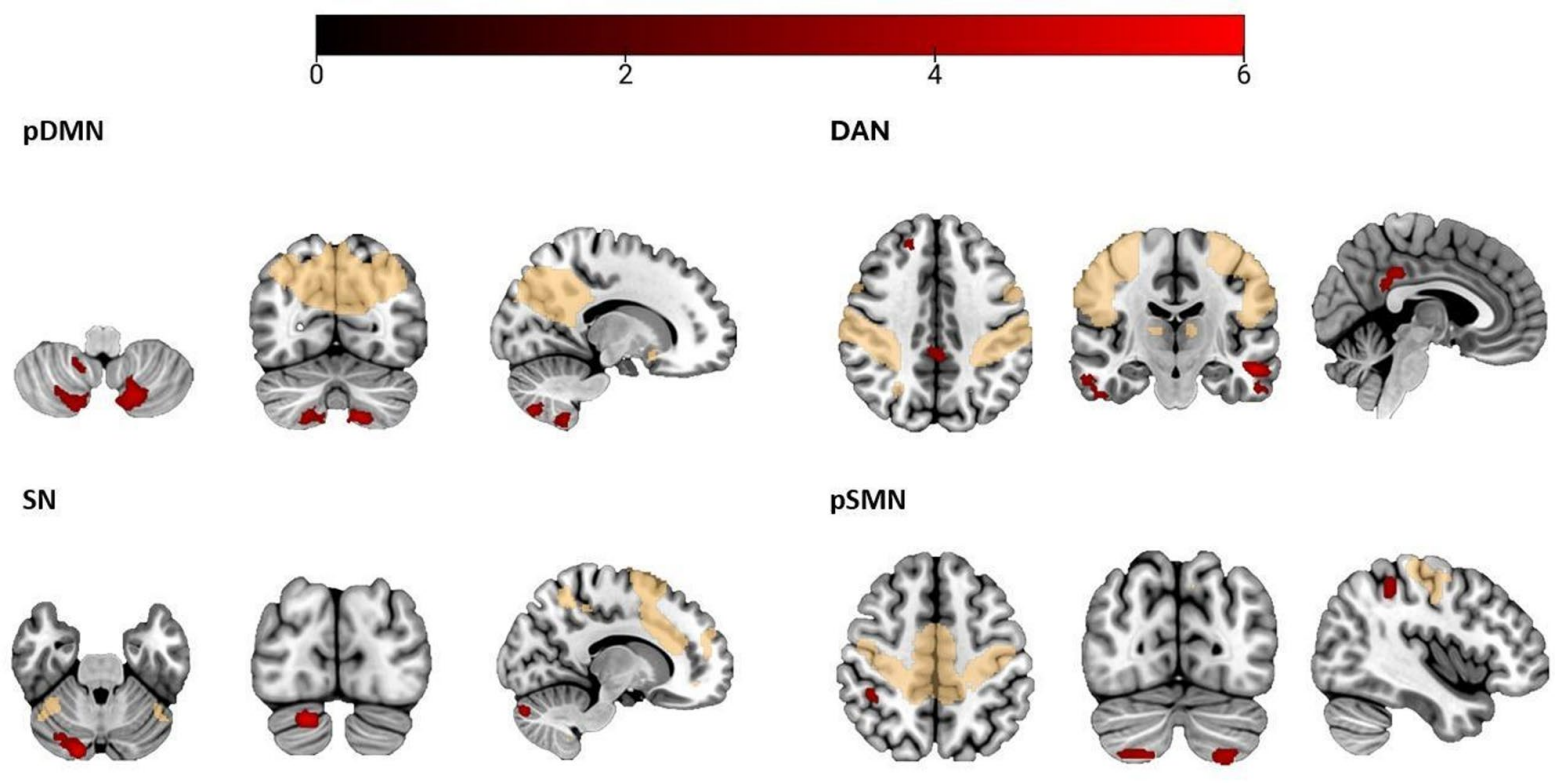
Regions showing greater ICA-derived rs-FC in IPVAW survivors compared to non-victims. Note. Red: Clusters showing greater ICA-derived rs-FC in IPVAW survivors compared to non-victims; Yellow: whole-sample maps of each network. pDMN: Posterior default network, SN: Salience network, DAN: Dorsal attention network, pSMN: Primary sensorimotor network

**Fig. 3:**
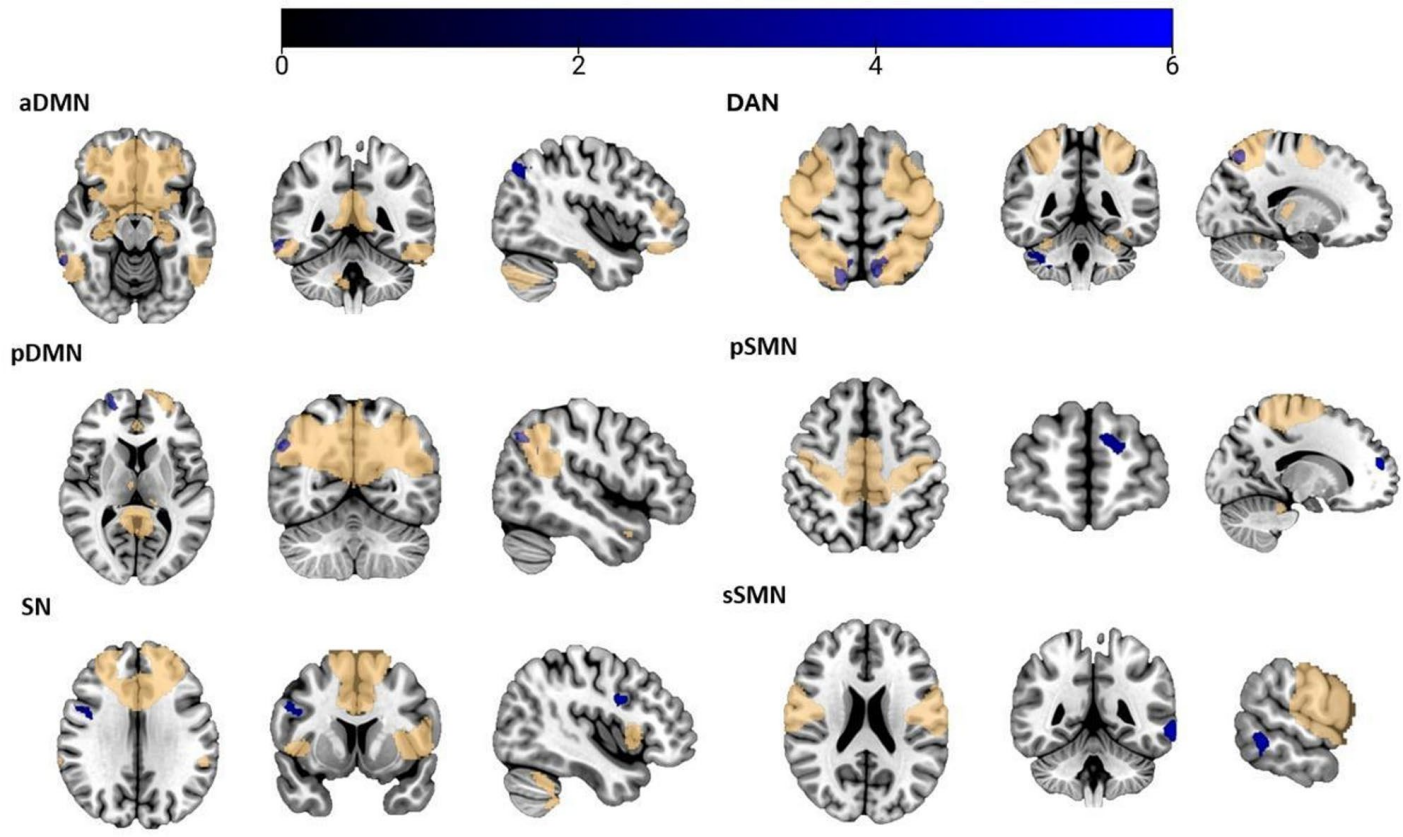
Regions showing reduced ICA-derived rs-FC in IPVAW survivors compared to non-victims. Note. Blue: Clusters showing reduced ICA-derived rs-FC in IPVAW survivors compared to non-victims. Yellow: whole-sample maps of each network. aDMN: Anterior default network, pDMN: Posterior default network, SN: Salience network, DAN: Dorsal attention network, pSMN: Primary sensorimotor network, sSMN: Secondary sensorimotor network

**Fig. 4:**
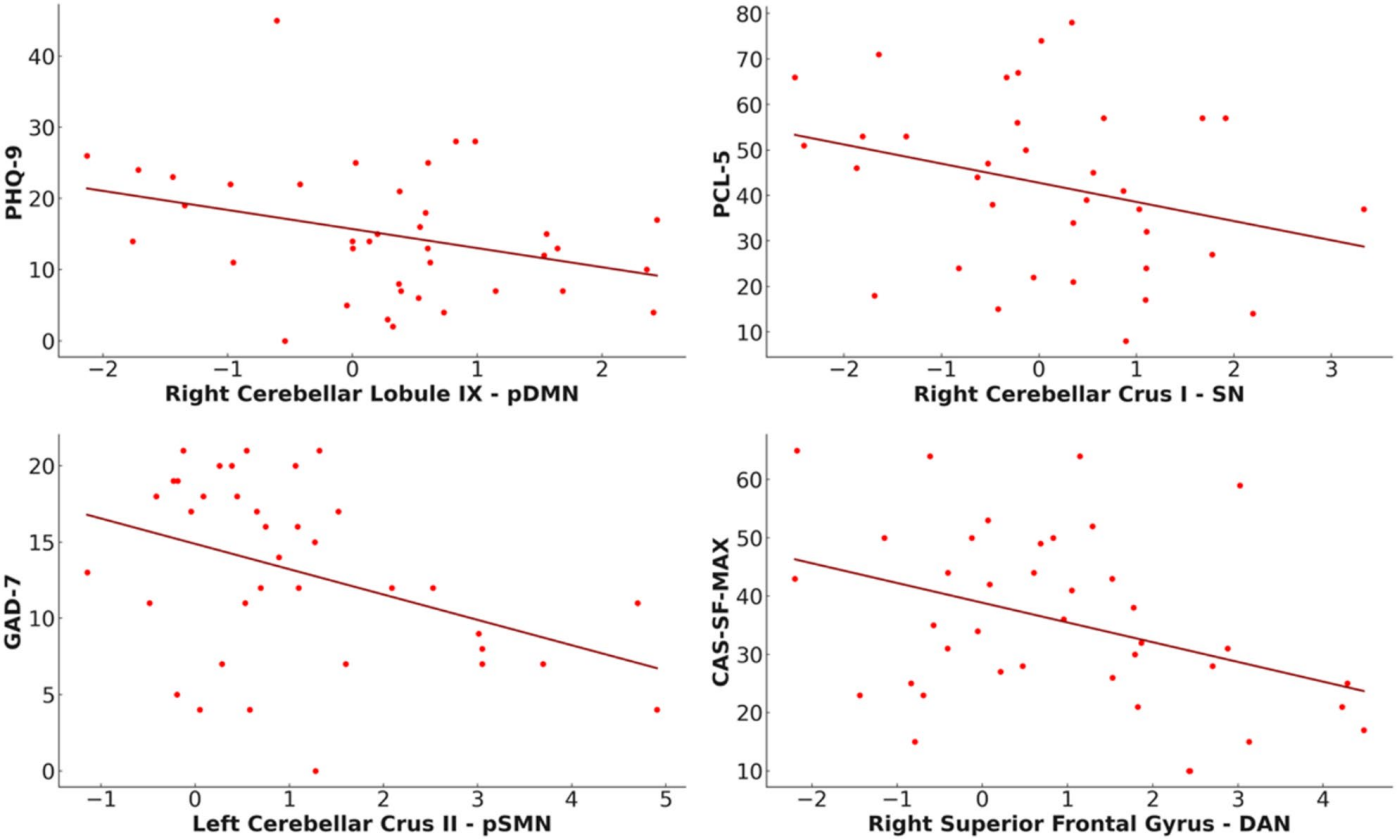
Relationship between specific greater ICA-derived rs-FC patterns in IPVAW group and clinical and severity of violence variables. Note. PHQ-9: Patient Health Questionnaire Depression Subscale, GAD-7: Generalized Anxiety Disorder, PCL-5: PTSD checklist for DSM-5, CAS-SF-MAX: Maximum score obtained in Composite Abuse Score, pDMN: posterior default network, pSMN: primary sensory network, SN: salience network, DAN: dorsal attention network

**Fig. 5:**
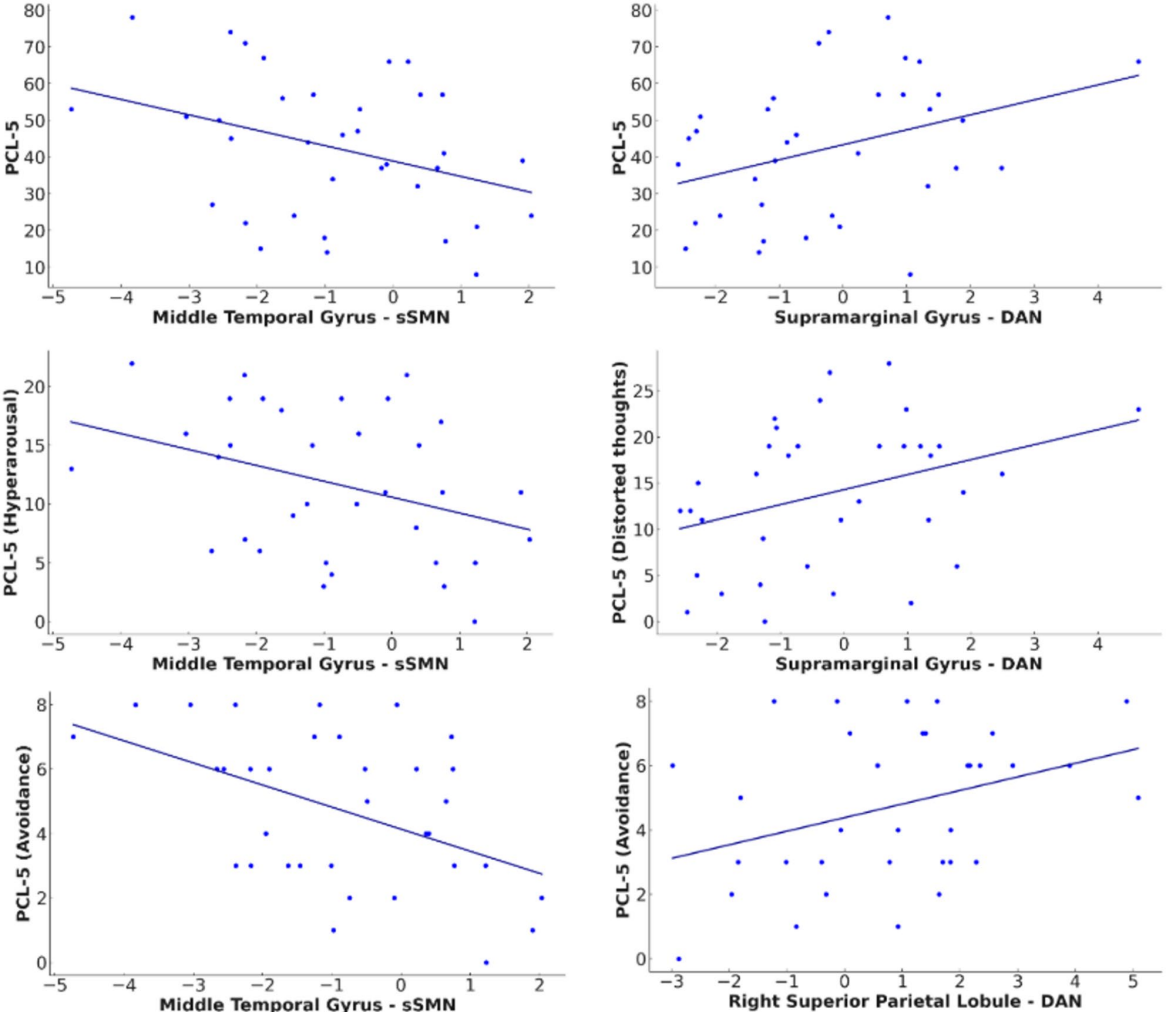
Relationship between specific reduced ICA-derived rs-FC patterns in IPVAW group and clinical variables. Note. PCL-5: PTSD checklist for DSM-5, sSMN: Secondary sensorimotor network, DAN: Dorsal attention network

Results are organized by network. Neuroimaging findings are also divided into inside- and outside-network connectivity, followed by their clinical-connectivity associations within the IPVAW group. Although none of the correlations remain significant after Bonferroni correction, we report all the correlational results at an uncorrected significance level of 0.05. Results are presented in Table 3; Figs. 2, 3, 4 and 5.

**Table 3:** Between-group differences in ICA-derived rs-FC

		X	Y	Z	Cluster size	T
ICs	Survivors > Non-victims					
pDMN	Left cerebellar lobule VIII	-16	-70	-52	279	4.91
	Right cerebellar lobule VIII	16	-74	-52	213	4.61
	Right cerebellar lobule IX	12	-56	-58	95	4.43
SN	Right cerebellar Crus I	16	-84	-28	205	5.27
pSMN	Left cerebellar Crus II	-26	-72	-56	472	4.88
	Right cerebellar lobule VIIb /Crus II	18	-76	-52	150	4.47
	Right inferior parietal lobule	42	-40	52	105	3.97
DAN	Left mid temporal gyrus	-54	18	-16	275	5.76
	Right inferior temporal gyrus	54	-24	-30	181	4.80
	Right superior frontal gyrus	18	46	44	177	4.60
	Mid-posterior cingulate cortex	8	-38	34	318	4.59

	**Survivors < Non-victims**					
pDMN	Right angular gyrus	54	-64	36	118	4.71
	Right superior frontal gyrus	20	58	6	106	4.40
aDMN	Right mid temporal gyrus	62	-46	-14	91	4.37
	Right angular gyrus	42	-70	42	197	4.32
SN	Right frontal inferior gyrus	44	6	28	89	3.98
sSMN	Left mid temporal gyrus	-66	-44	-6	133	4.76
pSMN	Left superior frontal gyrus	-16	54	16	110	5.16
DAN	Right superior parietal lobule	14	-68	60	255	5.25
	Left precuneus	-8	-62	62	135	4.52
	Right cerebellar Crus I	38	-44	-36	103	4.65
	Left supramarginal gyrus	66	-32	34	102	4.40

*ICs* Independent components, *aDMN* Anterior default mode network, *pDMN* Posterior default mode network, *SN* Salience network, *FPN* Frontoparietal network, *pSMN* Primary sensorimotor network, *sSMN* Secondary sensorimotor network, *DAN* Dorsal attention network

#### Anterior Default Mode Network (aDMN)

Inside the network, IPVAW group exhibited less connectivity in the right middle temporal gyrus (rMTG) compared to non-victims.

When examining results outside the network, the IPVAW group showed less connectivity in the right angular gyrus (rAG) compared to the non-victims control group.

#### Posterior Default Mode Network (pDMN)

Inside the network, IPVAW group exhibited less connectivity in the right angular gyrus compared to non-victims.

When examining the results outside the network, IPVAW group exhibited greater connectivity in several bilateral posterior cerebellum regions. More concretely, in the left cerebellar lobule VIII, right cerebellar lobule VIII, and right cerebellar lobule IX. The IPVAW group also showed less connectivity in the right superior frontal gyrus compared to non-victims.

In addition, clinical-connectivity associations within the IPVAW group, showed that the right cerebellar lobule IX connectivity was negatively correlated with PHQ-9 scores (*r* = − .330, *p* = .043).

#### Salience Network (SN)

No between-group differences were found inside the network. 

When examining results outside the network, the IPVAW group showed greater connectivity in right cerebellar Crus I and less connectivity in the right frontal inferior gyrus compared to non-victims.

Moreover, the right cerebellar Crus I connectivity showed a negative correlation with scores in the hyperarousal subscale of the PCL-5 *(r* = − .409, *p* = .020) within the IPVAW survivors’ group.

#### Frontoparietal Network (FPN)

No significant group differences were observed inside or outside the network.

#### Primary Sensorimotor Network (pSMN)

Connectivity inside the network did not show significant differences between groups.

When examining results outside the network, the IPVAW group showed less connectivity in the left superior frontal gyrus. Moreover, the IPVAW group also showed greater connectivity in the right inferior parietal lobule and in the bilateral posterior cerebellum, more concretely in left cerebellar Crus II, and in right cerebellar lobule VIIb /Crus II.

Additionally, clinical-connectivity associations within the IPVAW group showed a negative correlation between connectivity in left cerebellar Crus II and GAD-7 scores (*r* = − .339, *p* = .019).

#### Secondary Sensorimotor Network (sSMN)

No between-group differences were found inside the network.

When examining results outside the network, the IPVAW group showed reduced connectivity in the left middle temporal gyrus compared to non-victims.

Additionally, the left middle temporal gyrus connectivity was negatively correlated with total PCL-5 score (*r* = − .354, *p* = .047), as well as with the hyperarousal *(r* = − .355, *p* = .046) and the avoidance subscales (*r* = − .523, *p* = .002) within the IPVAW group.

#### Dorsal Attention Network (DAN)

Inside the network, IPVAW group showed less connectivity in the right superior parietal lobule and left precuneus.

In terms of outside-network results, the IPVAW group showed less connectivity in the right cerebellar Crus I and supramarginal gyrus, and greater connectivity in the left middle temporal gyrus, right inferior temporal gyrus, right superior frontal gyrus, and mid-posterior cingulate gyrus.

Moreover, clinical-connectivity associations showed that the right superior frontal gyrus was negatively correlated with CAS-SF-MAX (*r* = − .429, *p* = .009). The left supramarginal gyrus was positively correlated with total PCL-5 score *(r* = .374, *p* = .035), and with the PCL-5 distorted thoughts subscale (*r* = .392, *p* = .027). The right superior parietal lobule was positively correlated with the PCL-5 avoidance subscale (*r* = .352, *p* = .048).

### Follow-up analyses: results after controlling for adverse childhood experiences

When controlling for ACE scores, the analyses showed that the between-group differences found across all networks (i.e., aDMN, pDMN, SN, FPN, SMN, and DAN) remain consistent.

Regarding the clinical-connectivity correlations, all results remained consistent, except for the association between the right cerebellar lobule IX (pDMN) and PHQ-9 score, which was no longer significant (*r* = –.337, *p* = .051).

## Discussion

To date, only three studies have examined brain connectivity in IPVAW survivors, all of which used connectivity-based methods such as seed-based functional analyses [15] or graph-theoretical analyses for structural connectivity [38]. These studies focused on examining specific connectivity alterations, leaving the intrinsic organization of large-scale resting-state networks largely unexplored. The present study expands on this literature by applying ICA to characterize the intrinsic brain architecture of IPVAW survivors by comparing RSNs between women survivors and non-victimized women. At first glance, the overall findings indicate that connectivity differences are balanced between the two groups. That is, we cannot assert a general pattern of hyper- or hypoconnectivity across the networks analyzed in IPVAW survivors, highlighting the need to examine differences at a finer scale. A closer look reveals that IPVAW survivors exhibited less rs-FC within the DAN and DMN. Beyond this within-network pattern, most significant differences emerged in outside-network connectivities, showing both increased and decreased rs-FC. This pattern supports previous studies emphasizing the importance of inter-network analyses in understanding the complexity of brain function in IPVAW survivors [16]. Of note, a consistent greater outside-network connectivity involving the cerebellum was observed in several networks (i.e., DMN, SMN, and SN). Although exploratory in nature, this cerebellar pattern was also repeatedly found associated with clinical symptoms in our sample, suggesting the cerebellum’s potential involvement in understanding IPVAW’s impact on the intrinsic brain connectivity. However, these associations did not survive correction for multiple comparisons and should therefore be interpreted with caution.

In terms of inside-network findings, the IPVAW group exhibited hypoconnectivity within both the DAN and DMN, with no differences observed in other relevant networks such as the SN or FPN. On one hand, the reduced connectivity within the DAN aligns with previous findings in adult populations with histories of childhood violence, where they also found the same connectivity pattern [60]. Given DAN’s role in supporting goal-directed attention and voluntary focus, reduced connectivity within this network may underlie difficulties with sustained attention, greater distractibility, and impaired filtering of irrelevant stimuli, core features captured by the avoidance subscale [61]. Although the correlation was opposite to what we expected, connectivity of the right superior parietal lobule, a core region of the DAN, was associated with scores on the avoidance subscale of the PTSD measure. This finding tentatively suggests a link between altered connectivity of this DAN region and PTSD symptomatology.

On the other hand, the reduced connectivity within the DMN in IPVAW survivors aligns with previous studies conducted in the same population. Despite the use of different methodological approaches, these studies have also reported reduced connectivity in specific DMN nodes, such as the precuneus [16] and the isthmus cingulate [15]. In addition, a prior structural connectivity study in IPVAW reported increased involvement of other DMN-related regions, such as the middle temporal gyrus [38], suggesting a different connectivity pattern than our results. While in the present study we did not find an association between within-DMN connectivity and clinical symptoms, previous research involving populations that have suffered other forms of violence (i.e., adolescents that suffered childhood violence and war veterans) [33, 62, 63] suggests a similar pattern of less within DMN-connectivity. Considered together, and acknowledging the differences between the populations, this pattern of reduced connectivity involving the DAN and the DMN could represent brain reorganization in response to chronic interpersonal violence. Future studies are needed to determine its specificity, clinical relevance, and potential trait-like nature [60, 64].

Our results showed no significant inside-network differences in the remaining networks of interest. Despite previous connectivity studies in IPVAW population [15, 16, 38] reported altered connectivities of regions within the SN such as anterior insula [16] and anterior cingulate cortex [38], and within the FPN, including the superior parietal and frontal cortices [15], we did not observe these patterns in the present study.

Regarding outside-network connectivity results, our study found a consistent pattern among IPVAW survivors of greater connectivity of the cerebellum in the pDMN, SN, and pSMN networks. Crucially, this cerebellar hyperconnectivity was associated with lower levels of depression, PTSD, and anxiety symptoms respectively, suggesting a potential compensatory mechanism. Nonetheless, as these correlations did not remain significant after Bonferroni correction, they should be viewed as preliminary until replicated in larger studies. The cerebellum has traditionally been known for its role in motor control; however, accumulating evidence indicates its integral involvement in higher cognitive and affective functions through widespread connectivity with cortical and subcortical regions [65, 66]. Specifically, the cerebellum contributes to global brain processing, including emotional regulation, attention, and sensorimotor integration [67]. In the context of trauma and psychopathology, cerebellar hyperconnectivity to large-scale networks such as the DMN and SN may reflect a functional adaptation aimed at maintaining cognitive and emotional stability despite the burden of stress-related neural disruptions [68]. This compensatory role could facilitate enhanced modulation of internally directed thoughts (DMN), detection of salient stimuli and arousal (SN), and sensorimotor coordination (SMN). Future longitudinal studies are needed to determine whether this cerebellar engagement represents an adaptation or a dynamic response to trauma [69, 70].

In addition to the greater cerebellar connectivity across networks, several specific outside-network connectivity differences between groups were also observed. For instance, women exposed to IPVAW exhibited reduced rs-FC between the sSMN and the left middle temporal gyrus (MTG) compared to non-victims. This reduced connectivity was associated in our data with more PTSD symptomatology, particularly, avoidance symptoms, and alterations in arousal and reactivity. The sSMN integrates bodily sensations with action, while the MTG is involved in episodic memory, processing meaning, and making contextual associations with past experiences [71]. Our results align with previous findings in individuals with PTSD, which also reported reduced connectivity between the sSMN and the MTG. Although speculative, authors have suggested that this altered connectivity may relate to trauma-linked avoidance and disruptions in integrating interoceptive signals with autobiographical memory, which could in turn be associated with heightened arousal/reactivity [45, 72, 73].

Another key outside-network outcome reveals that IPVAW survivors exhibit greater connectivity in the DAN with regions that are classically considered part of the DMN, namely, the mid-posterior cingulate gyrus and the middle temporal gyrus. Normally, the DAN and DMN operate in an antagonistic manner: when one network is activated, the other tends to deactivate [74]. In the IPVAW group, this greater connectivity between the DAN and DMN regions could reflect reduced functional segregation between these two networks which has been suggested to play a role in conflict between internally and externally directed attention, making it more difficult to disengage from internal thoughts and focus on the external environment [75, 76].

A final result from the data shows that the greater outside-network connectivity between the DAN and the superior frontal gyrus was found to be associated with lower severity of experienced violence. Although this finding runs contrary to our hypothesis and we cannot explain it a priori, it is possible that other variables related to the violence could have been overlooked. Examples include the duration of exposure to violence, the time elapsed since the violence ended, or the specific subtype of violence experienced (e.g., physical or sexual). Another explanation could be related to the possible impact of protective factors that may be acting as mediators between brain connectivity and mental health after suffering IPVAW, such as resilience. Exploring these nuances in future research could help clarify whether this result persists or not.

### Limitations

One limitation of our study is the relatively small sample size, which may affect the stability and future replicability of our findings. Women survivors from IPVAW are a difficult population to sample, as in our case, they had to be referred from women’s information centers linked with the Andalusian Women’s Institute. Additionally, it is challenging for them to adhere to a study that requires several days of evaluation, as they often have many responsibilities, such as being the sole caregivers for their children or having medical appointments or court dates. However, despite these practical limitations, the present study represents the largest sample size to date. Another potential limitation of our study was the disparity in education levels between the groups. To address this, we accounted for education level as a covariate in all neuroimaging and correlational analyses. A third limitation lies in the inability to assess the potential impact of TBI on brain connectivity. Although this variable was measured, the sample size of the potential subgroups of women with and without TBI were too small to allow for further statistical analyses. In addition, the authors acknowledge that the resting-state acquisition lasted approximately 6 min, which may limit the reliability and stability of the ICA-derived resting-state networks. Future studies should aim to replicate and extend the present findings using longer scan durations. Moreover, we were not able to include several potentially relevant clinical characteristics (e.g., borderline personality disorder, dissociative symptoms, other personality traits) that have been linked to both trauma-related mental health outcomes and adverse childhood experiences. As these variables were not assessed, we cannot rule out that the associations between IPVAW severity, psychopathology, and brain network connectivity are partly influenced or moderated by such unmeasured factors, rather than solely reflecting the impact of IPVAW. Finally, as stated in the results section, the correlations did not survive correction for multiple comparisons and therefore should be regarded as tentative and exploratory. Nevertheless, we believe that these findings provide valuable preliminary insights for further investigation.

## Conclusions

The present study demonstrates that survivors of IPVAW exhibit distinctive rs-FC patterns in core large-scale brain networks, including DMN, DAN, SN and SMN, when compared to non-victims. Specifically, survivors showed less within-network connectivity in the DMN and DAN, which may be associated with disruptions in internally and externally directed attention processes. In addition, outside-network connectivity results revealed altered connectivity profiles characterized by cerebellar hyperconnectivity with the DMN, SN, and SMN. Importantly, this greater cerebellar coupling was found associated with lower symptomatology of depression, PTSD, and anxiety, raising possible hypotheses of a compensatory or neuroprotective role of the cerebellum in the intrinsic brain architecture of IPVAW survivors.

### Social and clinical implications

Our findings help provide a better understanding of the lasting consequences experienced by women who have survived IPVAW. By exploring the intrinsic neural correlates that may underlie the daily challenges these women face—challenges that often go unrecognized in society—our study points to a possible hidden burden associated with this experience. Clinically, our results offer useful insights that might help guide the development of future rehabilitation approaches tailored to the needs of these survivors. Finally, the findings support the inclusion of functional brain assessments in clinical and hospital settings, not only for expanding our knowledge of trauma-related effects, but also for improving the medical care available to women survivors of IPVAW.

### Future research directions

Additional research is warranted to replicate our findings and to open new avenues for understanding the neural sequelae associated with IPVAW. Further work could explore other relevant brain networks by applying state-of-the art analysis methods (e.g., dynamic causal modeling). Of note, cerebellum showed to be a relevant and complex brain region that needs to be studied in depth due to its possible compensatory or neuroprotective role when experiencing interpersonal violence. Moreover, it is essential to explore potential protective factors such as resilience and their impact on the neural-psychopathological associations, and how subtypes of violence (e.g., physical, psychological, sexual, economic) may differentially affect brain function and therefore women’s daily life functioning. Upcoming research should also take into account relevant variables that can cause cerebral changes in this population such as cortisol levels, TBI, hypoxic/ischemic brain damage, and medical conditions related to IPVAW [77]. Through this multi-level research approach, we aim to support women survivors of IPVAW, promote their well-being, and advocate for a more egalitarian society.

## Supplementary Information

Supplementary Material 1.

## Data Availability

The datasets used and/or analysed during the current study are available from the corresponding author on reasonable request.

## Abbreviations

Acronym: Full expression
ACE: Adverse childhood experiences
aDMN: Anterior default mode network
AUDIT: Alcohol Use Disorders Identification Test
BISA: Brain Injury Severity Assessment
CIMCYC: Mind, Brain, and Behavior Research Center
DAN: Dorsal attention network
DMN: Default mode network
FOV: Field of View
FPN: Frontoparietal network
FWHM: Full-width-at-half-maximum
ICA: Independent component analysis
ICs: Independent components
IPV: Intimate partner violence
IPVAW: Intimate partner violence against women
MNI: Normalization in the Montreal Neurological Institute
MTG: Middle temporal gyrus
pDMN: Posterior default mode network
pSMN: Posterior sensorimotor network
PTSD: Posttraumatic stress disorder
rAG: Right angular gyrus
rs-FC: Resting-state functional connectivity
rs-fMRI: Resting-state functional magnetic resonance imaging
RSNs: Resting-state networks
SD: Standard Deviation
SMN: Sensorimotor network
SN: Salience network
sSMN: Secondary sensorimotor network
TBI: Traumatic brain injury
TE: Echo time
TR: Repetition time
