# Supplementary material for: Altered resting-state functional connectivity in women survivors of intimate partner violence: an ICA study
Source: BMC Psychol. 2026 Mar 20;14:371. doi: 10.1186/s40359-026-04063-x (PMC13005384; doi:10.1186/s40359-026-04063-x)
Supplement: Supplementary file 1 — Supplementary Material 1. [file 40359_2026_4063_MOESM1_ESM.docx]

**SUPPLEMENTARY MATERIAL**

**Participants**

The inclusion criteria stipulated that both groups of participants must be at least 18 years old. Exclusion criteria for both groups included a pre-participation evaluation that assessed (a) age-related cognitive decline for participants over 50, indicated by a score below 27 on the Mini Mental State Examination (MMSE; 1), (b) severe TBI unrelated to IPV, defined as a loss of consciousness for over 30 minutes and/or post-traumatic amnesia for over 24 hours following an impact to the head, in accordance with ACRM criteria (2) (c) recent or habitual use of illegal psychoactive substances, (d) lack of Spanish language proficiency, (e) claustrophobia, (f) age above 65, (g) the presence of internal metallic objects (such as dental or intrauterine devices) that could be incompatible with magnetic resonance equipment, (h) body volume exceeding the MRI scanner's dimensions.

In addition, given the high prevalence of IPVAW and its frequent underreporting (3, 4), we assessed potential experiences of IPVAW also, in the self-identified as non-victim participants. For this, all interested participants completed the Composite Abuse Scale-Short Form (CAS-SF; 5) to assess potential experiences of physical, psychological, and/or sexual violence from current or former partners. In addition, while the CAS-SF typically assesses violence over the past year, this study extended its focus to IPVAW throughout the participants' lifetime. Since a Spanish version of the CAS-SF was unavailable, we adapted and translated the measure according to International Test Commission guidelines (6), as detailed in (7). Furthermore, the authors generated a new measure—CAS-SF-MAX—which reflected the highest score of violence reported either in the past year or prior to it, allowing us to obtain a maximum violence value that did not exclude the severity of violence experienced by women who had suffered it either recently or in the more distant past. Participants reporting any instance of physical or sexual violence (e.g., hitting or strangulation) were excluded from the non-victim group. Potential non-victims who reported psychological violence occurring more often than "sometimes" were also excluded. Additionally, non-victims who experienced isolated instances of psychological violence (“once” or “sometimes”) within the last five years were removed if their total score exceeded five. For example, if a participant experienced five single instances of psychological violence more than five years ago, she was retained in the non-victim group. However, if these instances occurred within the last five years, she was excluded. Applying these criteria, one participant was removed from the non-victim group.

Regarding alcohol use among participants in both groups, only cases of alcohol dependence (as opposed to general alcohol consumption) were grounds for exclusion. Specifically, scores greater than 15 in the Alcohol Use Disorders Identification Test (AUDIT-C) were excluded (8). Women survivors with certain chronic health conditions, such as fibromyalgia, were not excluded due to the condition's high prevalence in this population (9) and its potential association with TBI and psychological trauma (10, 11). Sociodemographic and health-related information on age, education level, nationality, civil status, severity of IPV, adverse childhood experiences, and mental health (generalized anxiety, depression, and PTSD) are displayed in Table 1.

***Violence and Mental Health Assessment***

***Severity of IPV***

The Composite Abuse Scale (Revised) - Short Form (CASR-SF) (5) is a 15-item self-report tool designed to evaluate the severity and intensity of intimate partner violence experienced over the past 12 months. This scale captures the frequency of psychological, physical, and sexual violence on a 0–5 scale, with a total possible score ranging from 0 to 75 (where a higher score indicates greater severity of violence). The original CASR-SF demonstrated strong internal consistency (0.942) (5) and was translated into Spanish for use in this study, adhering to the International Test Commission Guidelines for Translating and Adapting Tests, Second Edition (ITC, 6). To account for women who experienced violence over longer periods but not within the past year, the measure was also applied retrospectively for earlier periods, allowing for an assessment of lifetime IPV types and frequencies. To account for women who experienced violence over longer periods but not within the past year, the CASR-SF was also administered retrospectively to capture exposure prior to the past 12 months. For each participant, we selected the highest psychological, physical and sexual severity of violence score obtained from the past 12-month assessment and the prior-to-12-months assessment, referred to as CAS-MAX, providing a comprehensive measure of the most severe IPVAW experienced.

***Possible TBI and Strangulation***

The evaluation of potential TBI was conducted using the Brain Injury Severity Assessment (BISA) semi-structured interview, devised by (12). This interview incorporates the criteria outlined by the American Committee for Mild Traumatic Brain Injury (13) to define mild TBI. While the criteria for severe TBI are clear-cut (e.g., unconsciousness lasting beyond 30 minutes or post-traumatic amnesia lasting more than 24 hours after the incident), the definition of mild TBI is less straightforward. As per the American Committee for Mild Traumatic Brain Injury (Special Interest Group of the American Congress of Rehabilitation Medicine), it is characterized by:

Any instance of unconsciousness, memory loss for events before or after the accident, or changes in mental state at the accident time (e.g., feeling dazed, disoriented, or confused); presence of focal neurologic deficit(s), which may be transient. However, the injury severity should not surpass the following criteria: unconsciousness lasting approximately 30 minutes or less, an initial Glasgow Coma Scale score of 13–15 after 30 minutes, and post-traumatic amnesia not exceeding 24 hours (13).

Due to the retrospective nature of the BISA interview (12), typically conducted weeks or months post-traumatic event, the Glasgow score was not evaluated. Nevertheless, all other alterations in consciousness, as per this definition, were examined in the interview concerning violence inflicted by an intimate partner (e.g., strangulation attempts, mild and severe TBI resulting from hits to the head or face). Changes in consciousness were identified as symptoms encompassing feelings of dizziness, being stunned, disorientation, seeing "stars or spots," losing consciousness or fainting, or struggling to recall events right before or after the violent incident. For instance, the initial question inquired, "After any actions by your partner, have you ever lost consciousness or blacked out?" If the participant confirmed such an experience, or any of the mentioned symptoms related to their partner's actions, follow-up questions delved into the cause (e.g., whether it involved a fist, object, shaking, or strangulation). Participants were also asked to estimate the time of the first and most recent incidents, along with the total number of occurrences. Eight out of the 39 participants from the survivor group were not able to complete the BISA interview. In consequence, the percentage of TBI in the survivor group was calculated with 31 participants.

***Adverse Childhood Events***

Participants completed the Adverse Childhood Experiences Questionnaire (ACE;14), which consists of 10 questions about traumatic experiences in childhood, such as abuse, neglect, and family-related challenges. The ACE yields a total score between 0 and 10 and demonstrates reliability, with an alpha of .61 for family issues and .80 for sexual abuse and overall score (15). One out of the 39 participants from the survivor group was not able to complete the ACE questionnaire.

***PTSD*** The PTSD Checklist (PCL-5; 16, 17, 18) is a 20-item questionnaire that assesses PTSD symptoms based on DSM-5 diagnostic criteria, covering intrusion, avoidance, cognitive changes, mood alterations, and hypervigilance. Survivors rated their symptoms in connection with intimate partner violence, while non-victims assessed symptoms related to other traumatic or stressful life events. Responses were recorded on a Likert scale from 0 (not at all) to 5 (extremely). Symptom severity was calculated by summing scores across all items, resulting in a total score between 0 and 80. In veteran studies, the PCL-5 has shown high internal consistency (α = .96), strong test-retest reliability (r = .84), and robust convergent and discriminant validity (17). Additionally, the PCL-5 has demonstrated solid psychometric properties in studies involving female survivors of intimate partner violence (α = .93; median inter-item r = .44) (19). Five out of the 39 participants from the survivor group, and 2 out of the 39 participants from the non-victims were not able to complete the PCL-5 questionnaire.

***Generalized Anxiety***

The Generalized Anxiety Disorder Questionnaire (GAD-7; 20) is a seven-item scale for detecting generalized anxiety. Participants rated the frequency of their anxiety symptoms over the past two weeks on a Likert scale ranging from 0 to 3 (0 = not at all, 1 = several days, 2 = more than half the days, 3 = nearly every day). Total scores, ranging from 0 to 21, are calculated by summing the scores across the seven items. In the Spanish population, the GAD-7 has demonstrated high sensitivity (88.8%) and specificity (94.4%) (21). Among women survivors, it has shown strong internal consistency (94%) (22). Two out of the 39 participants from the survivor group, and 2 out of the 39 participants from the non-victims were not able to complete the GAD-7 questionnaire.

***Depression***

Depressive symptoms were assessed using the Patient Health Questionnaire Depression Subscale (PHQ-9; 23), a nine-item questionnaire based on DSM-IV criteria for major depression. Participants rated each item on a Likert scale from 0 to 3 (0 = not at all, 1 = several days, 2 = more than half the days, 3 = nearly every day). The total PHQ-9 score, which ranges from 0 to 27, is obtained by summing scores across all nine items. The measure has shown a sensitivity of 84% and specificity of 72% in samples from 3,000 primary care and 3,000 obstetric and gynecological patients (24). One out of the 39 participants from the survivor group, and 2 out of the 39 participants from the non-victims were not able to complete the PHQ-9 questionnaire.

***Alcohol Consumption***

The Alcohol Use Disorders Identification Test (AUDIT) (8, 25) was developed by the World Health Organization (WHO) to identify risky drinking, alcohol abuse, or dependence. This self-administered screening test includes 10 questions: three on alcohol consumption, three on alcohol dependence, and three on problems related to alcohol use. Items are rated on a Likert scale from 0 to 4. The total score, ranging from 0 to 40, is calculated by summing the scores of all items. Among women, a score above 7 suggests a strong likelihood of alcohol-related harm, while a score above 20 suggests alcohol dependence (8, 25). Two out of the 39 participants from the survivor group were not able to complete the AUDIT questionnaire.

**References**

1. Lobo A, Ezquerra J. The mini-mental state examination: A simple test, practical, for alterations intelectivas in medical patients. Luso-Spanish Acts of Neurology and Psychiatry. 1979;3:189–202.
2. Committee on Mild Traumatic Brain Injury. Definition of mild traumatic brain injury. J Head Trauma Rehabil. 1993;8:48–59.
3. Goldin Y, Haag HL, Trott CT. Screening for history of traumatic brain injury among women exposed to intimate partner violence. PM R. 2016;8(11):1104–1110. doi:10.1016/j.pmrj.2016.05.006.
4. Zieman G, Bridwell A, Cárdenas JF. Traumatic brain injury in domestic violence victims: A retrospective study at the Barrow Neurological Institute. J Neurotrauma. 2017;34(4):876–880. doi:10.1089/neu.2016.4579.
5. Ford-Gilboe M, Wathen N, Varcoe C, MacMillan HL, Scott-Storey K, Mantler T, et al. Development of a brief measure of intimate partner violence experiences: The Composite Abuse Scale (Revised)-Short Form (CASR-SF). BMJ Open. 2016;6(12):e012824. doi:10.1136/bmjopen-2016-012824.
6. International Test Commission (ITC). ITC guidelines for translating and adapting tests (2nd ed.). Int J Test. 2018;18(2):101–134.
7. Daugherty JC, Verdejo-Román J, Pérez-García M, Hidalgo-Ruzzante N. Structural brain alterations in female survivors of intimate partner violence. J Interpers Violence. 2020;37(7–8):NP4684–NP4717. doi:10.1177/0886260520959621.
8. Bradley KA, Bush KR, Epler AJ, Dobie DJ, Davis TM, Sporleder JL, et al. Two brief alcohol screening tests from the Alcohol Use Disorders Identification Test (AUDIT): Validation in a female Veterans Affairs patient population. Arch Intern Med. 2003;163(7):821–829. doi:10.1001/archinte.163.7.821.
9. Chandan JS, Thomas T, Bradbury-Jones C, Russell R, Bandyopadhyay S, Nirantharakumar K, et al. Female survivors of intimate partner violence and risk of depression, anxiety and serious mental illness. Br J Psychiatry. 2019;:1–6. doi:10.1192/bjp.2019.124.
10. Afari N, Ahumada SM, Wright LJ, Mostoufi S, Golnari G, Reis V, et al. Psychological trauma and functional somatic syndromes: A systematic review and meta-analysis. Psychosom Med. 2014;76(1):2–11. doi:10.1097/PSY.0000000000000010.
11. Grandhi R, Tavakoli S, Ortega C, Simmonds M. A review of chronic pain and cognitive, mood, and motor dysfunction following mild traumatic brain injury: Complex, comorbid, and/or overlapping conditions? Brain Sci. 2017;7(12):160. doi:10.3390/brainsci7120160.
12. Valera EM, Berenbaum H. Brain Injury Severity Assessment (BISA) interview. University of Illinois; 1997.
13. Ruff RM, Iverson GL, Barth JT, Bush SS, Broshek DK, The NAN Policy and Planning Committee. Recommendations for diagnosing a mild traumatic brain injury: A National Academy of Neuropsychology education paper. Arch Clin Neuropsychol. 2009;24(1):3–10. doi:10.1093/arclin/acp006.
14. Felitti VJ, Anda RF, Nordenberg D, Williamson DF, Spitz AM, Edwards V, et al. REPRINT OF: Relationship of childhood abuse and household dysfunction to many of the leading causes of death in adults: The Adverse Childhood Experiences (ACE) Study. Am J Prev Med. 2019;56(6):774–786. doi:10.1016/j.amepre.2019.04.001.
15. Ford DC, Merrick MT, Parks SE, Breiding MJ, Gilbert LK, Edwards VJ, et al. Examination of the factorial structure of adverse childhood experiences and recommendations for three subscale scores. Psychol Violence. 2014;4(4):432–444. doi:10.1037/a0037723.
16. Blevins CA, Weathers FW, Davis MT, Witte TK, Domino JL. The PTSD Checklist for DSM-5 (PCL-5): Development and initial psychometric evaluation. J Trauma Stress. 2015;28(6):489–498. doi:10.1002/jts.22059.
17. Bovin MJ, Marx BP, Weathers FW, Gallagher MW, Rodriguez P, Schnurr PP, et al. Psychometric properties of the PTSD Checklist for DSM-5 (PCL-5) in veterans. Psychol Assess. 2016;28(11):1379–1391. doi:10.1037/pas0000254.
18. Wortmann JH, Jordan AH, Weathers FW, Resick PA, Dondanville KA, Hall-Clark B, et al. Psychometric analysis of the PTSD Checklist-5 (PCL-5) among treatment-seeking military service members. Psychol Assess. 2016;28(11):1392–1403. doi:10.1037/pas0000260.
19. Krause ED, Kaltman S, Goodman LA, Dutton MA. Longitudinal factor structure of posttraumatic stress symptoms related to intimate partner violence. Psychol Assess. 2007;19(2):165–175. doi:10.1037/1040-3590.19.2.165.
20. Spitzer RL, Kroenke K, Williams JB, Löwe B. A brief measure for assessing generalized anxiety disorder: The GAD-7. Arch Intern Med. 2006;166(10):1092–1097. doi:10.1001/archinte.166.10.1092.
21. Garcia-Campayo J, Zamorano E, Ruiz MA, Pardo A, Perez-Paramo M, Lopez-Gomez V, et al. Cultural adaptation into Spanish of the Generalized Anxiety Disorder-7 (GAD-7) scale as a screening tool. Health Qual Life Outcomes. 2010;8(1):8. doi:10.1186/1477-7525-8-8.
22. Do KN, Weiss B, Pollack A. Cultural beliefs, intimate partner violence, and mental health functioning among Vietnamese women. Int Perspect Psychol. 2013;2(3):149–163. doi:10.1037/ipp0000004.
23. Kroenke K, Spitzer RL. The PHQ-9: A new depression diagnostic and severity measure. Psychiatr Ann. 2002;32(9):509–515. doi:10.3928/0048-5713-20020901-06.
24. Kroenke K, Spitzer RL, Williams JB. The PHQ-9: Validity of a brief depression severity measure. J Gen Intern Med. 2001;16(9):606–613. doi:10.1046/j.1525-1497.2001.016009606.x.
25. Bush K, Kivlahan DR, McDonell MB, Fihn SD, Bradley KA. The AUDIT alcohol consumption questions (AUDIT-C): An effective brief screening test for problem drinking. Arch Intern Med. 1998;158(16):1789–1795. doi:10.1001/archinte.158.16.1789.
